# Supplementary material for: Transcriptomic response of the picoalga Pelagomonas calceolata to nitrogen availability: new insights into cyanate lyase function
Source: Microbiol Spectr. 2025 Mar 25;13(5):e02654-24. doi: 10.1128/spectrum.02654-24 (PMC12054182; doi:10.1128/spectrum.02654-24)
Supplement: Figures S1 and S2 — Pelagomonas growth curves and gene expression levels between triplicates. [file spectrum.02654-24-s0001.pdf]

Supplementary information

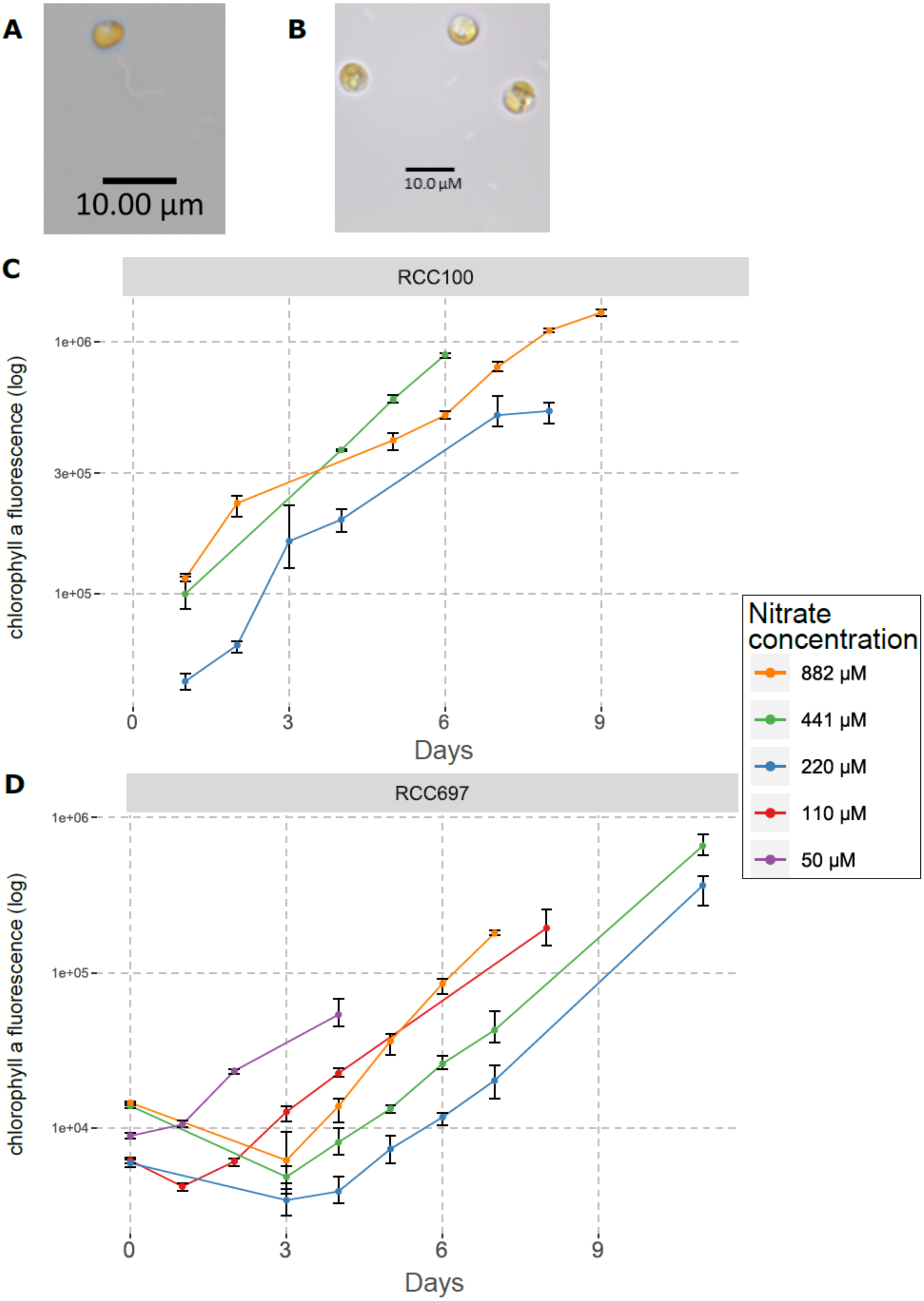

Supplementary Figure 1: A) *P. calceolata* RCC100, light microscopy. B) *P. calceolata* RCC697, light microscopy. C and D) *P. calceolata* RCC100 (C) and RCC697 (D) growth with different nitrate concentrations. The chlorophyll a fluorescence is used as a proxy for cell concentration (arbitrary unit). *P. calceolata* cells were harvested for RNA extraction at the last time point of each condition.

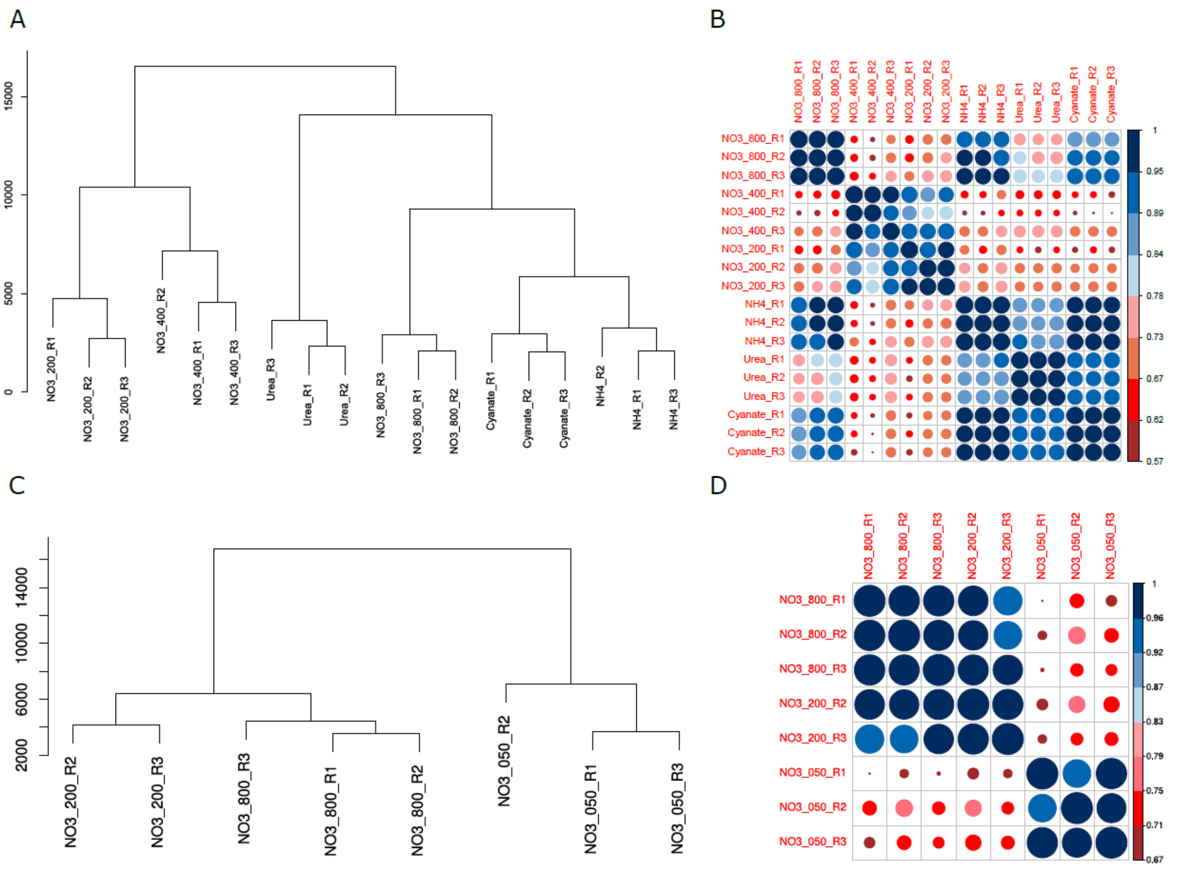

Supplementary Figure 2: Gene expression levels of *P. calceolata* RCC100 and RCC697 under different nitrate concentrations. A and C) Hierarchical clustering of the gene expression levels of *P. calceolata* RCC100 (A) and RCC697 (C). B and D) Pearson's r correlations of gene expression levels between each pair of samples for *P. calceolata* RCC100 (B), and RCC697 (D). Each replicate is processed independently and is indicated by R1 to R3 for each condition
